# Population structure at different minor allele frequency levels

**DOI:** 10.1186/1753-6561-8-S1-S55

**Published:** 2014-06-17

**Authors:** Omar De la Cruz, Paola Raska

**Affiliations:** 1 Department of Epidemiology and Biostatistics, Case Western Reserve University School of Medicine, 10900 Euclid Ave, Cleveland, OH 44106, USA

## Abstract

Inferring population genetic structure from large-scale genotyping of single-nucleotide polymorphisms or variants is an important technique for studying the history and distribution of extant human populations, but it is also a very important tool for adjusting tests of association. However, the structures inferred depend on the minor allele frequency of the variants; this is very important when considering the phenotypic association of rare variants.

Using the Genetic Analysis Workshop 18 data set for 142 unrelated individuals, which includes genotypes for many rare variants, we study the following hypothesis: the difference in detected structure is the result of a "scale" effect; that is, rare variants are likely to be shared only locally (smaller scale), while common variants can be spread over longer distances. The result is similar to that of using kernel principal component analysis, as the bandwidth of the kernel is changed. We show how different structures become evident as we consider rare or common variants.

## Background

Inferring population genetic structure from large-scale genotyping of single-nucleotide polymorphisms (SNPs) or variants (SNVs), often performed using principal component analysis (PCA) [1] or model-based clustering [2], is an important technique for studying the history and distribution of extant human populations [3], but it is also a very important tool for adjusting tests of association [1,4].

Thanks to the increasing availability of sequencing technology, it is possible now to identify very rare variants and to type them on large samples of individuals, extending the reach of the genome-wide association study design. However, methods for detecting population structure and for adjusting association tests accordingly, should take into account the fact that the population structures inferred depend on the minor allele frequency (MAF) of the SNVs; this is very important when considering the phenotypic association of rare variants [5].

In this article we show evidence of different structures at different MAF levels. We propose that the difference is a result of a "scale" effect: rare variants are likely to be shared only locally (smaller scale), whereas common variants can be spread over longer distances. The result is similar to that of using kernel principal component analysis (KPCA) [6] because the bandwidth (ie, scale) of the kernel is changed (De la Cruz and Susan Holmes, work in preparation). This similarity between the behavior of PCA at different MAF levels and KPCA at different scales is further evidence, albeit circumstantial, of the connection between MAF levels and scale.

Using the Genetic Analysis Workshop 18 (GAW18) data set for 142 unrelated individuals, which includes genotypes for many rare variants, we show how different structures become evident as we consider rare or common variants and how these structures transform smoothly as we change the window of allowed MAF values. We suggest that such a procedure provides a more complete picture of the structure of the population.

## Methods

We selected at random a set of 82,594 SNVs from the odd-numbered autosomal chromosomes. The set is thin enough that linkage disequilibrium caused solely by proximity along the chromosome does not affect the results substantially. We dropped 133 SNVs that are monomorphic for the set of unrelated individuals, leaving 82,461 SNVs. We did not filter out those variants that appear in only 1 individual, even though they are less informative about the relationships between different individuals. It is important, though, to consider the number of such private variants for each individual, as that might add a linear dimension related to the total number of private variants. (*Private *here refers to a variant that appears only once in the sample of 142 individuals. More of these variants can only make an individual more different from the others, whereas a shared variant can make 2 individuals more alike.)

We sorted the variants by MAF and selected a sliding window of 900 SNVs, going from the rarest variants to the most common. We used a total of 533 such windows, which is considered a dense enough concentration to make the continuity of the eigenvalues evident. Because these windows overlapped substantially, the principal component analyses performed on consecutive windows are closely related.

The computation of the principal components was performed via the singular value decomposition, after centering and scaling [7].

**Figure 1:**
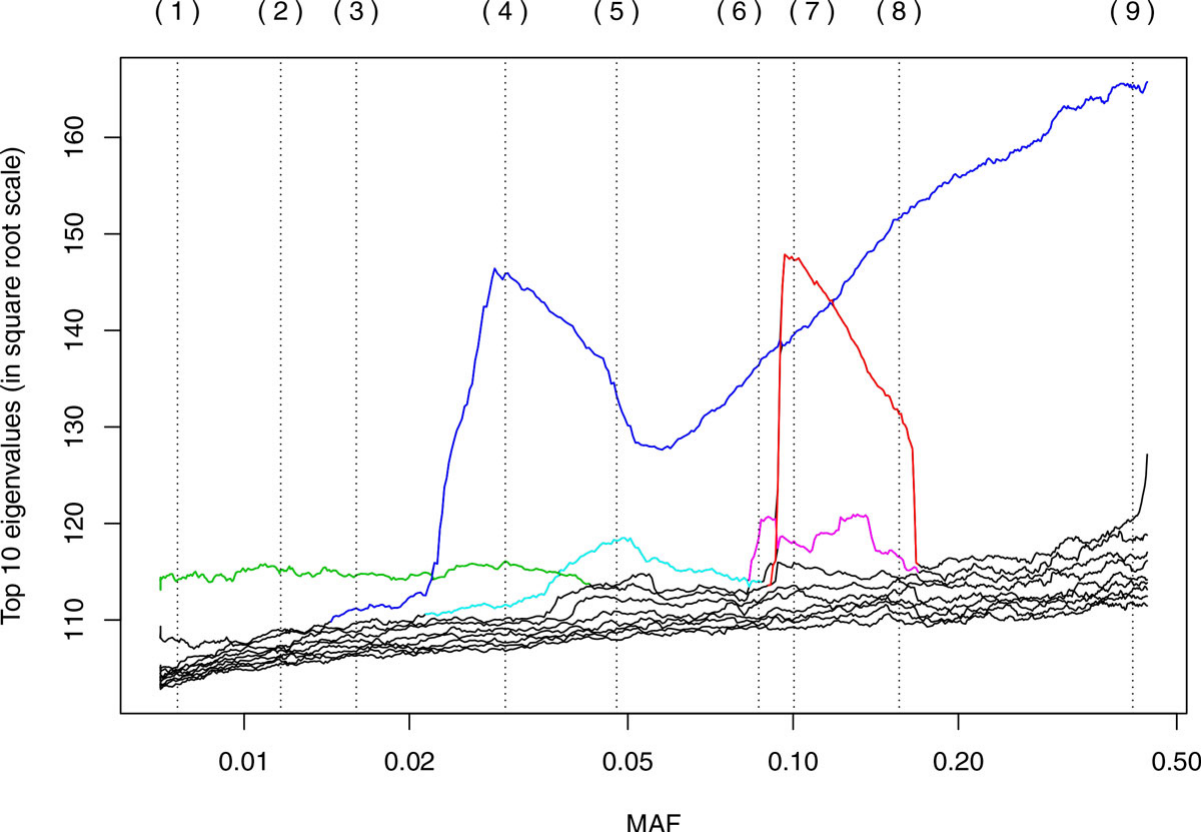
**Eigenvalues for different values of the MAF**. Each curve shows the evolution of an eigenvalue as we slide the window of SNVs used in PCA. The colors were selected manually to show likely continuation whenever there is a crossing of eigenvalues. For clarity, the plot shows only 10 eigenvalues. The vertical dotted lines correspond to the locations that were selected for the scatterplot panels in Figure 2.

**Figure 2:**
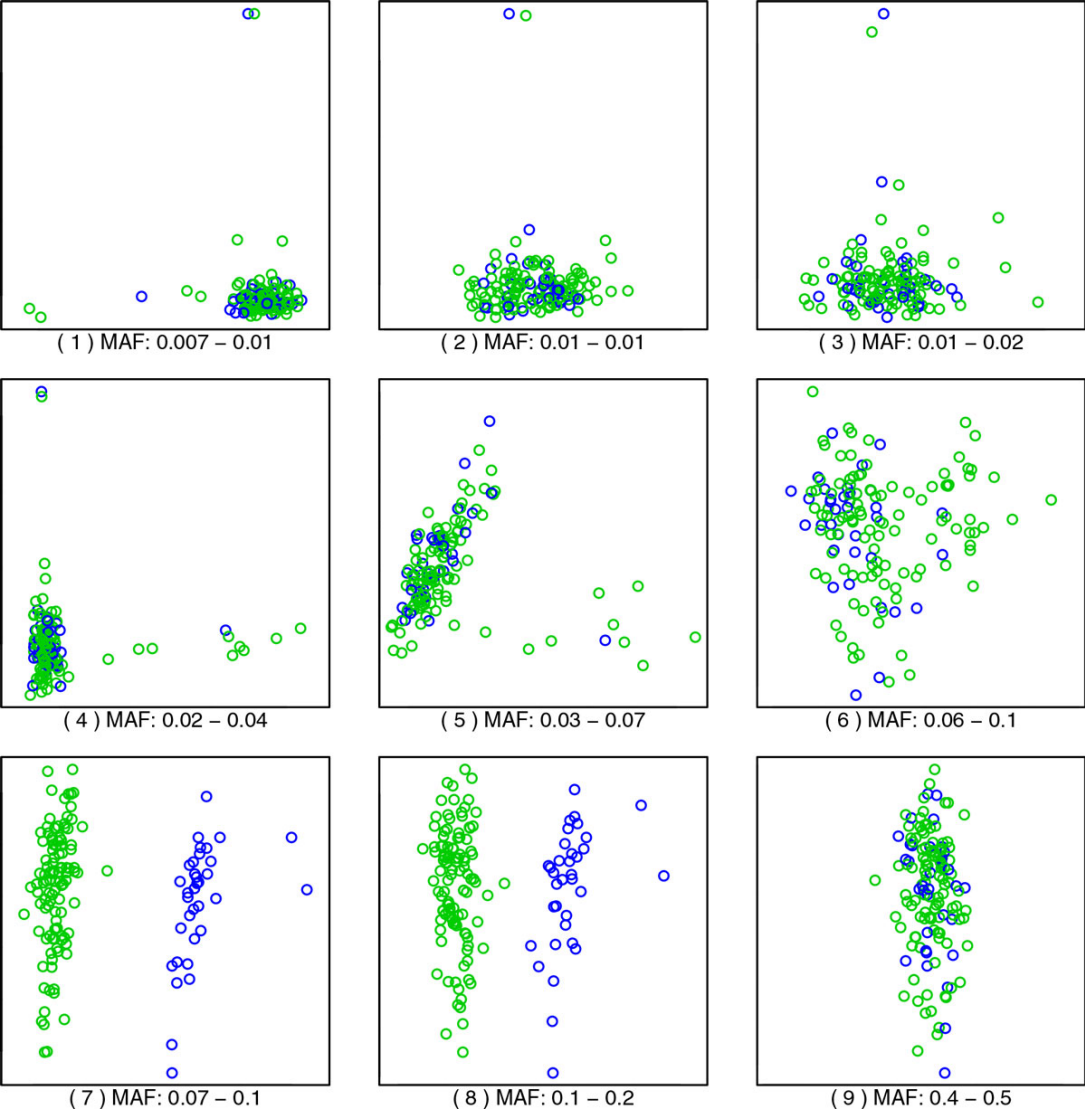
**Scatterplot of the 2 top principal components, for different values of the MAF**. The range of MAF of the 9000 SNVs used is spelled out below each panel; also, the numbers in parenthesis match the vertical lines in Figure 1. The colors are based on the clusters that appear in panes (7) and (8). Other panels mix these colors, so the cluster information is lost. Notice that only 2 components are used to facilitate visualization; other components also contain information.

Coloring of eigenvalue paths in Figure 1 was done manually, and the color was extended only as far as it seemed clearly defined. In Figure 2, some manual rotations (changing signs, which are arbitrary for eigenvectors, or switching principal component [PC]1 and PC2) were used to make the relationship between panels clearer.

The theoretical argument for the persistence of the eigenvalue-eigenvector relationships as the window slides is given by perturbation theory [8]: Because 2 contiguous windows share a large number of markers, the corresponding variance-covariance matrices are close to each other; consequently, small perturbations of the matrix lead to a small change in the eigenvalues and eigenvectors. The behavior is potentially more complicated when eigenvalues cross, because at the crossing point, by definition, there are repeated eigenvalues. However, our experiments suggest that the matching persists even after such crossings in many cases.

## Results

Figure 1 shows how different eigenvalues increase and decrease in value as the MAF changes. In reality, the eigendecompositions are computed independently, and it is difficult to track automatically how each eigenvalue/eigenvector evolves as MAF changes, but from the plot it is clear that different features become more important at different values of MAF. This is similar to what is observed when the bandwidth (scale) of a kernel is changed in KPCA. Figure 2 contains 9 scatterplots of the top 2 principal components; these panels are labeled (1) to (9), and the corresponding location in the MAF scale is marked by vertical lines in Figure 1. Each panel corresponds to the best 2-dimensional representation of the genetic relationships between the unrelated individuals. As we show here, these relationships are different depending on whether we use rare variants (small MAF) or more common variants. The MAF increases from panel to panel, by rows. (The reversions in the plots correspond to the arbitrary signs for the eigenvectors in an eigendecomposition.) Notice how the structure in the first panel is transformed into something different as we progress through the panels.

For MAF values below 0.02, the dominant PCA features are pairs of points that are set out from the rest (see the Discussion for an explanation). One pairs is picked out by a component that corresponds to the eigenvalue path colored in green in Figure 1; that pair can be easily located at the top of panels (1) through (4) in Figure 2.

For MAF values above 0.02, the dominant feature corresponds to the eigenvalue path labeled in blue, except for an eigenvalue path that suddenly rises to prominence at MAF 0.09, even surpassing the blue curve briefly, and disappears equally suddenly at MAF 0.15 (colored in red). The "blue" eigenvalues correspond to a continuous axis, represented vertically in panels (6) through (9), while the "red" eigenvalues capture a clustering in the population into 2 clusters (plus 2 outliers). The colors in Figure 2 reflect this clustering; see the Discussion for further details on this phenomenon.

## Discussion

Panel (1) in Figure 2, corresponding to a MAF of 0.7% to 1% (rare variants), shows a structure that is commonly seen in PCA plots of genotype data: "flares" that extend from a central position. There are 3 clusters, with 1 staying at the center and the other 2 radiating away, with some individuals in the middle. As argued in the previously mentioned work in progress by De la Cruz and Holmes, this is likely related to the diversity in each of the clusters. As one considers more common SNVs, that is, higher values of MAF, a different structure arises in panels (6) through (8), a structure that corresponds to the rise of the "red" eigenvalue in Figure 1, showing 2 clusters. Finally, a dominating linear dimension, together with an orthogonal but smaller dimension, appears toward the values of MAF of 40% to 50% (common variants). Notice that we use only 2 components to facilitate visualization; other components can contain important information, too, and the common practice is to use the top 10 PCs when adjusting for population structure.

The flares in panels (1) through (4) are dominated by a pair of individuals at the tip. These are set out from the rest of the group not because their genotype is different, but because they are more closely related to each other than to the rest. In other words, each of these "groups of 2" have reduced diversity, compared with the overall group. Each of these pairs form a feature that becomes more important when observed at a smaller scale. This corresponds to the appropriate eigenvalues rising to the top, and this pattern is evident in Figure 1.

As argued by Mathieson and McVean [5], it can be problematic to perform a PCA-based adjustment for an association test on rare alleles when the structure is computed using common alleles. It is also likely that a population structure estimated using a mix of common and rare alleles will just mix the signals, diluting both types of structures. Our analysis also shows that relying on rare alleles tends to pick up the more closely related pairs of individuals, which is unlikely to be useful in adjusting for population structure.

**Figure 3:**
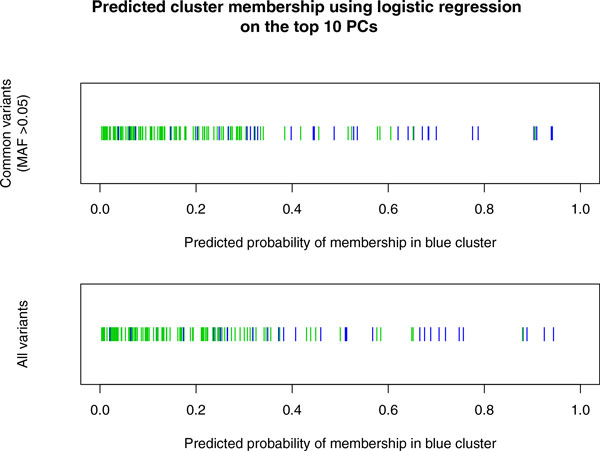
**Attempt to recover the clusters observed for MAF between 0**.09 and 0.15 (see Figure 1, panels 7 and 8) by means of a single PCA analysis using all the SNVs (*bottom panel*) or all the common variants (SNVs with MAF >0.05, *top panel*). We performed logistic regression using cluster membership as the response, and using the top 10 PCs as the predictors. The separation of the clusters is very poor, indicating that the top 10 PCs commonly used in genome-wide association studies in adjusting for population structure fail to uncover the clustering observed using narrow frequency windows.

The most intriguing feature to come out of our analysis is the separation in clusters that happens for values of MAF between 0.09 and 0.15. This feature is still present at other nearby frequencies, but the corresponding eigenvalue drops fast below the others. This clustering is not an artifact of the SNVs selected: When using a denser panel extracted from chromosome 11, the same feature arises, in the same furtive way (data not shown). It should be noted that this clustering does not become apparent when performing PCA using a panel of SNVs of all MAFs, or when restricted to common variants (MAF >0.05). Indeed, even logistic regression using the top 10 PCs does a poor job of replicating the separation of the clusters (Figure 3). In other words, our multifrequency analysis (which can be considered multiscale) uncovers important features that a single mixed-frequency analysis misses.

We tried to match the cluster to the available phenotypic data, but none of the following factors matched: sex, status as sequenced versus genotyped-by-chip-plus-imputation, or pedigree membership. (Because this sample was obtained from an admixed population, it is possible that this phenomenon is a consequence of admixture; however, we do not have data on the ancestry proportions of the individuals.) Thus, the true nature of the clustering remains a mystery and should be taken into account when analyzing the data for disease associations, possibly by including a component in the adjustment that separates the clusters, or by checking any potential discoveries *a posteriori *for unequal distribution between the 2 clusters.

This is an interesting question: If two individuals share a very rare variant, not only are they likely to be from the same locality, but they are also likely to be somewhat related. If this is the case, they will tend to share a higher proportion of common variants than other pairs of individuals. Why then would an analysis based on common variants not give the same information as one based on rare variants? A set of top PCs derived from common markers and the whole sample would pick up only large, continent-wide trends. A PC might pick up local correlation, but it would have a small eigenvalue.

There is an important consequence of the local nature of the components obtained from rare variants: many components might be needed to fully describe the population structure at the given scale. As an illustration, consider the following situation: Two components, derived from common markers, can be enough to capture the main genetic geographical structure on a continent (say, a north-south component and an east-west component). However, if we use components derived from rare alleles, being able to discriminate between neighboring villages, we would need a large number of components to distinguish all the villages. Thus, the following recommendation can be made: Instead of incorporating a large number of PCs derived from rare variants into the regression tests of association, one should check any discoveries *a posteriori *for the possibility of spurious association with one or more of those PCs.

## Conclusions

We believe that a considerable amount of information can be gained by exploring the population structure at different values of MAF. Using rarer variants corresponds to looking at structures that arise at smaller scales, because rare variants are likely to be shared between individuals located near each other, whereas common variants can be shared at longer distances. We posit that population genetic structure is a multiscale phenomenon, and that to elucidate behaviors at different scales, it is useful to consider sets of variants grouped by MAF.

## Competing interests

The authors declare that they have no competing interests.

## Authors' contributions

ODC and PR designed the overall study, ODC conducted statistical analyses and drafted the manuscript. All authors read and approved the final manuscript.
